# MicroRNAs Differentially Regulate Carbonyl Reductase 1 (CBR1) Gene Expression Dependent on the Allele Status of the Common Polymorphic Variant rs9024

**DOI:** 10.1371/journal.pone.0048622

**Published:** 2012-11-01

**Authors:** James L. Kalabus, Qiuying Cheng, Javier G. Blanco

**Affiliations:** Department of Pharmaceutical Sciences, The State University of New York at Buffalo, Buffalo, New York, United States of America; University of North Carolina at Chapel Hill, United States of America

## Abstract

MicroRNAs (miRNAs) are small RNAs responsible for the post-transcriptional regulation of a variety of human genes. To date, their involvement in the regulation of *CBR1* is unknown. This study reports for the first time the identification of microRNA-574-5p (hsa-miR-574-5p) and microRNA-921 (hsa-miR-921) as two miRNAs capable of interacting with the 3′-untranslated region (3′-UTR) of the *CBR1* gene and downregulating *CBR1* expression. Furthermore, we demonstrate that a common single-nucleotide polymorphism (SNP) in the *CBR1* 3′-UTR (rs9024, *CBR1* 1096G>A) differentially impacts the regulation of *CBR1* by hsa-miR-574-5p and hsa-miR-921 dependent on genotype. First, four candidate miRNAs were selected based on bioinformatic analyses, and were tested in Chinese hamster ovary (CHO) cells transfected with *CBR1* 3′-UTR constructs harboring either the G or A allele for rs9024. We found that hsa-miR-574-5p and hsa-miR-921 significantly decreased luciferase activity in CHO cells transfected with the *CBR1* 3′-UTR construct carrying the major rs9024 G allele by 35% and 46%, respectively. The influence of these miRNAs was different in cells transfected with a *CBR1* 3′-UTR construct containing the minor rs9024 A allele in that only hsa-miR-574-5p had a demonstrable effect (i.e., 52% decrease in lucifersase activity). To further determine the functional effects of miRNA-mediated regulation of polymorphic *CBR1*, we assessed CBR1 protein expression and CBR1 enzymatic activity for the prototypical substrate menadione in human lymphoblastoid cell lines with distinct rs9024 genotypes. We found that hsa-miR-574-5p and hsa-miR-921 significantly decreased CBR1 protein (48% and 40%, respectively) and CBR1 menadione activity (54% and 18%, respectively) in lymphoblastoid cells homozygous for the major rs9024 G allele. In contrast, only hsa-miR-574-5p decreased CBR1 protein and CBR1 activity in cells homozygous for the minor rs9024 A allele, and did so by 49% and 56%, respectively. These results suggest that regulation of human *CBR1* expression by hsa-miR-574-5p and hsa-miR-921 depends upon rs9024 genotype status.

## Introduction

MicroRNAs (miRNAs) are a family of small (∼22 nt), highly conserved, non-protein coding RNAs that have been recognized as important gene expression regulators [1], [2], [3]. It is estimated that the human genome contains around 1000 miRNA genes, and that each miRNA gene harbors the flexibility to potentially interact with multiple genes [4]. MiRNAs have been linked to the post-transcriptional regulation of a diverse host of genes that participate in the majority of important biological functions [5], [6], [7]. This class of RNAs regulates gene expression through partial, complementary Watson-Crick base-pairing of target mRNAs in the 3′-untranslated region (3′-UTR) of the message.

Carbonyl reductase 1 (CBR1) is a ubiquitous, cytosolic, short-chain dehydrogenase that catalyzes the two-electron reduction of a broad range of endogenous and xenobiotic compounds [8]. Among its most relevant pharmacological substrates are the antipsychotic haloperidol and the chemotherapeutic anthracyclines doxorubicin and daunorubicin [9], [10]. Recently, we documented extensive variability in *CBR1* expression in human liver [11] and heart [12] tissues. For example, normalized hepatic *CBR1* mRNA expression was found to vary almost 50-fold while cardiac *CBR1* mRNA expression levels varied 13-fold. Similarly, wide variability was found in CBR1 protein levels determined by quantitative immunoblotting (7-fold and 8-fold in liver and heart tissues, respectively). In line, and most therapeutically relevant, is the reported variability in CBR1 activity for the reduction of anthracycline substrates. For example, Gonzalez et al. and Kassner et al. found that hepatic CBR1 activity for the anthracycline substrate doxorubicin varied by approximately 22-fold [11], [13]. Functional single-nucleotide polymorphisms (SNPs) on the *CBR1* gene may impact *CBR1* expression and CBR1 activity. For instance, we reported that a relatively common SNP in the 3′-UTR of *CBR1*, *CBR1* 1096G>A (rs9024; *q*≈12.5% in Europeans and≈30% in Asians), impacts CBR1 phenotypes. In liver, homozygosis for the major G allele was associated with significantly higher CBR1 protein expression and CBR doxorubicin reductase activity [11]. We hypothesized that rs9024 might influence the binding of miRNAs to the 3′-UTR of *CBR1* and represent another example of miRNA pharmacogenomics [14].

To date, there are no reports of functional miRNA interactions with human *CBR1*. Therefore, our primary objective was to determine if any miRNAs are capable of regulating *CBR1*. First, we set out to identify miRNAs that might bind to the 3′-UTR of *CBR1 in silico*. Next, we tested candidate miRNA binding with human *CBR1* 3′-UTR constructs in cultures of the Chinese hamster ovary-derived cell line (CHO). To confirm the potential of the miRNA candidates to regulate polymorphic *CBR1*, we assessed CBR1 protein levels and CBR enzymatic activity in human lymphoblastoid cell lines with distinct rs9024 genotype status. Finally, we assessed the co-expression of the miRNA candidates and *CBR1* in human liver and heart tissue.

## Materials and Methods

### Ethics statement

The Health Sciences Institutional Review Board of the State University of New York at Buffalo has determined that this research project does not involve human subjects as defined under HHS regulations 45 CFR 46.102 (f).

### Bioinformatics

The full 3′-UTR sequence of human *CBR1* (GenBank sequence NM_001757.2) was retrieved using Entrez (http://www.ncbi.nlm.nih.gov/Entrez/). Potential miRNA binding sites along the *CBR1* 3′-UTR were found by comparing the results from multiple bioinformatic prediction databases including PITA [15], TargetScan [16], Microcosm Targets (formerly miRBase Targets) [17], [18], RNAhybrid [19], and PolymiRTS [20], [21].

### Cell culture and reagents

CHO-K1 cells (Chinese hamster ovary-derived cell line, CCL-61) were obtained from the American Type Culture Collection (Manassas, VA). Common cell culture reagents were purchased from Invitrogen (Carlsbad, CA). CHO-K1 cells were routinely cultured in 10 cm^2^ tissue culture dishes using RPMI 1640 medium supplemented with 10% (v/v) heat-inactivated fetal bovine serum (Sigma-Aldrich, St. Louis, MO), 100 U/ml penicillin, and 100 µg/ml streptomycin. Cultures were grown and maintained at low passage numbers (n<12) using standard incubator conditions of 37°C, 5% CO_2_, and 95% relative humidity.

Human lymphoblastoid cell lines (Coriell Institute, Camden, NJ) were routinely cultured in 75 cm tissue culture flasks using RPMI 1640 medium supplemented with 15% (v/v) heat-inactivated fetal bovine serum (Sigma-Aldrich, St. Louis, MO), 100 U/ml penicillin, and 100 µg/ml streptomycin. Suspension cultures were maintained in standard incubator conditions of 37°C, 5% CO_2_, and 95% relative humidity. These particular cell lines originate from the International HapMap project, a collaborative effort to create a haplotype map of the human genome, and were chosen for their utility in identifying genetic determinants of variability in gene expression [22], [23].

### Constructs

MicroRNA mimics and hairpin inhibitors for microRNA-574-5p (hsa-miR-574-5p), microRNA-656 (hsa-miR-656), microRNA-877-5p (hsa-miR-877-5p), and microRNA-921 (hsa-miR-921), as well as mimic and inhibitor negative controls, were obtained from Dharmacon (Chicago, IL). Full-length *CBR1* 3′-UTR constructs, one containing the G allele of *CBR1* 1096G>A (pCBR1-1096G) and the other containing the A allele (pCBR1-1096A), were synthesized by OriGene (Rockville, MD). The 3′-UTR fragments were cloned into a pMirTarget vector (OriGene) downstream of the firefly luciferase gene. All inserts were verified by direct DNA sequencing.

### Transfection

CHO-K1 cells were plated in 48-well plates 24 to 48 hours prior to transfection. CHO-K1 cells were co-transfected with *CBR1* 3′-UTR luciferase reporter constructs (500 ng) plus internal control plasmid pRL-TK (50 ng) and 15 nM miRNA mimic (hsa-miR-574-5p, hsa-miR-656, hsa-miR-877-5p, or hsa-miR-921) in the presence or absence of 5 nM specific miRNA inhibitor using DharmaFECT Duo transfection reagent (Dharmacon). Identical concentrations of miRNA mimic and inhibitor negative controls were also used. 24 hours post-transfection, cultures were washed once with phosphate-buffered saline solution and cells were lysed in freshly diluted passive lysis buffer (100 µl/well; Promega, Madison, WI) by incubating the plates at room temperature on a shaker at 200 rpm for 60 minutes. Luciferase reporter gene activities were determined with the Dual-Luciferase Reporter Assay System (Promega) per the manufacturer's instructions. Light intensity was measured in a Synergy HT luminometer equipped with proprietary software for data analysis (BioTek, Winooski, VT). Light intensity values from cell cultures transfected with *CBR1* 3′-UTR luciferase reporter constructs and *Renilla* luciferase control plasmids were used to correct for background. Corrected firefly luciferase activities were normalized to *Renilla* luciferase activities and expressed as fold increases with respect to the values obtained from control transfections with miRNA mimic negative control. Three independent experiments were performed in triplicate to evaluate reproducibility.

Lymphoblastoid cells were plated in 48-well plates 24 hours prior to transfection. Cells were co-transfected with 15 nM miRNA mimic (hsa-miR-574-5p, hsa-miR-656, hsa-miR-877, or hsa-miR-921) and miRNA mimic negative control in the presence or absence of 5 nM of specific miRNA inhibitor using the Neon transfection system (Invitrogen). 48 hours post-transfection, cells were pelleted for cytosolic protein extraction.

### Genotyping of lymphoblastoid cells for *CBR1* 1096G>A genotype status

The *CBR1* 1096G>A polymorphism was investigated by allelic discrimination with fluorescent probes and real-time PCR (Assays-by-Design; Applied Biosystems, Carlsbad, CA). Genotyping reactions were run as described [11], [12]. DNA samples of known rs9024 genotype status were used as positive controls.

### Quantitative immunoblotting of CBR1 in lymphoblastoid cells

To assess the influence of microRNA binding on CBR1 protein expression, lymphoblastoid cells (≈1×10^6^ cells) were incubated for 24 hours with 15 nM miRNA mimic in the presence or absence of 5 nM specific hairpin inhibitor. Cytosolic protein was isolated using the Minute Cytosolic and Nuclear Extraction kit (Invent Biotechnologies, Eden Prairie, MN) according to the manufacturer's instructions. CBR1 protein levels were quantitated essentially as described [24]. In brief, lymphoblast cytosols (35 µg) were loaded into 12% precast polyacrylamide gels (Invitrogen, Carlsbad, CA) and separated by electrophoresis. Protein blots were probed with a specific polyclonal anti-human CBR1 antibody (1∶1,000; Santa Cruz Biotechnology, Inc., Santa Cruz, CA) or anti-human β-actin antibody (1∶1,000; Santa Cruz Biotechnology, Inc.) and a secondary goat anti-rabbit IgG antibody conjugated with horseradish peroxidase (1∶10,000; Sigma-Aldrich). Immunoreactive bands were visualized with the ECL plus western blotting detection system (GE Healthcare, Chalfont St. Giles, UK). CBR1 band intensity values (pixels/mm^2^) were quantified with a ChemiDoc XRS gel documentation system (Bio-Rad Laboratories). Cytosolic CBR1 levels were calculated relative to the band density of cells transfected with miRNA mimic negative control.

### RNA degradation analysis

48 hours post-transfection with microRNA mimics and/or inhibitors, lymphoblastoid cells were treated with 10 µM actinomycin D (Enzo Life Sciences, Farmingdale, NY) to block *de novo* RNA synthesis. Cells were collected after 0, 2, 4, and 8 hours of actinomycin D incubation. Treatments were conducted in triplicate. *CBR1* mRNA levels were quantitated as described [11]. Briefly, total RNA (75 ng) from lymphoblastoid cells was reverse-transcribed and amplified using a one-step QuantiTect SYBR Green RT-PCR kit (Qiagen, Valencia, CA) with the following primers: 5′-TCAAGCTGAAGTGACGATGA-3′ (forward) and 5′-GGTGCACTCCCTTCTTTGTA-3′ (reverse). *CBR1* mRNA levels were determined by the comparative quantitation method. Individual *β-actin* mRNA levels were used for normalization. Experimental samples and standards for calibration curves were analyzed in quadruplicate. The relative amount of *CBR1* mRNA in each sample was automatically calculated with a comparative quantitation algorithm (iQ5 Optical System Software version 2.0, Bio-Rad, Hercules, CA). *CBR1* mRNA values were expressed relative to the normalized *CBR1* mRNA content of negative control-transfected cells.

### Detection of miRNA expression in human liver and heart tissue

The Institutional Review Board of the State University of New York at Buffalo approved this research. Human heart (left ventricle) and liver tissue samples from organ donors were procured from the National Disease Research Interchange (Philadelphia, PA) and the Liver Tissue Procurement and Distribution System (National Institutes of Health Contract N01-DK-9-2310), respectively. Cardiac and hepatic total RNA were extracted as previously described [11], [12]. To determine the gene expression of our candidate miRNAs in human tissues, we conducted real-time RT-PCR in triplicate in heart and liver total RNA (15 ng) using miScript Primer Assays (Qiagen). MiRNA expression levels in both tissue types were normalized to individual U6 gene levels and then expressed relative to corresponding amounts of hsa-miR-15a expression.

### Kinetic analysis

Maximal CBR1 enzymatic activities for the prototypical substrate menadione were measured in lymphoblastoid cell cytosols in a Synergy HT Multi-Detection Microplate Reader (Bio-Tek, Winooski, VT) [25], [26], [27]. Briefly, CBR1 enzymatic activity was assessed by measuring the oxidation rate of the CBR1 co-factor NADPH at 340 nm (molar absorption coefficient = 6220 M^−1^ cm^−1^). Reaction mixtures (0.25 ml) contained potassium phosphate buffer (0.1 M; pH 7.4), NADPH (200 µM; Sigma-Aldrich), and menadione (250 µM; Sigma-Aldrich) and were incubated at 37°C for 5 minutes. Kinetic reactions were started by the addition of cytosols (40 µl, total protein concentration: 1.1±0.4 mg/ml). Enzymatic velocities were automatically calculated by linear regression of the Δabsorbance/Δtime points with Bio-Tek's proprietary software for enzyme kinetic analysis.

## Results

### Bioinformatic analyses

**Figure 1:**
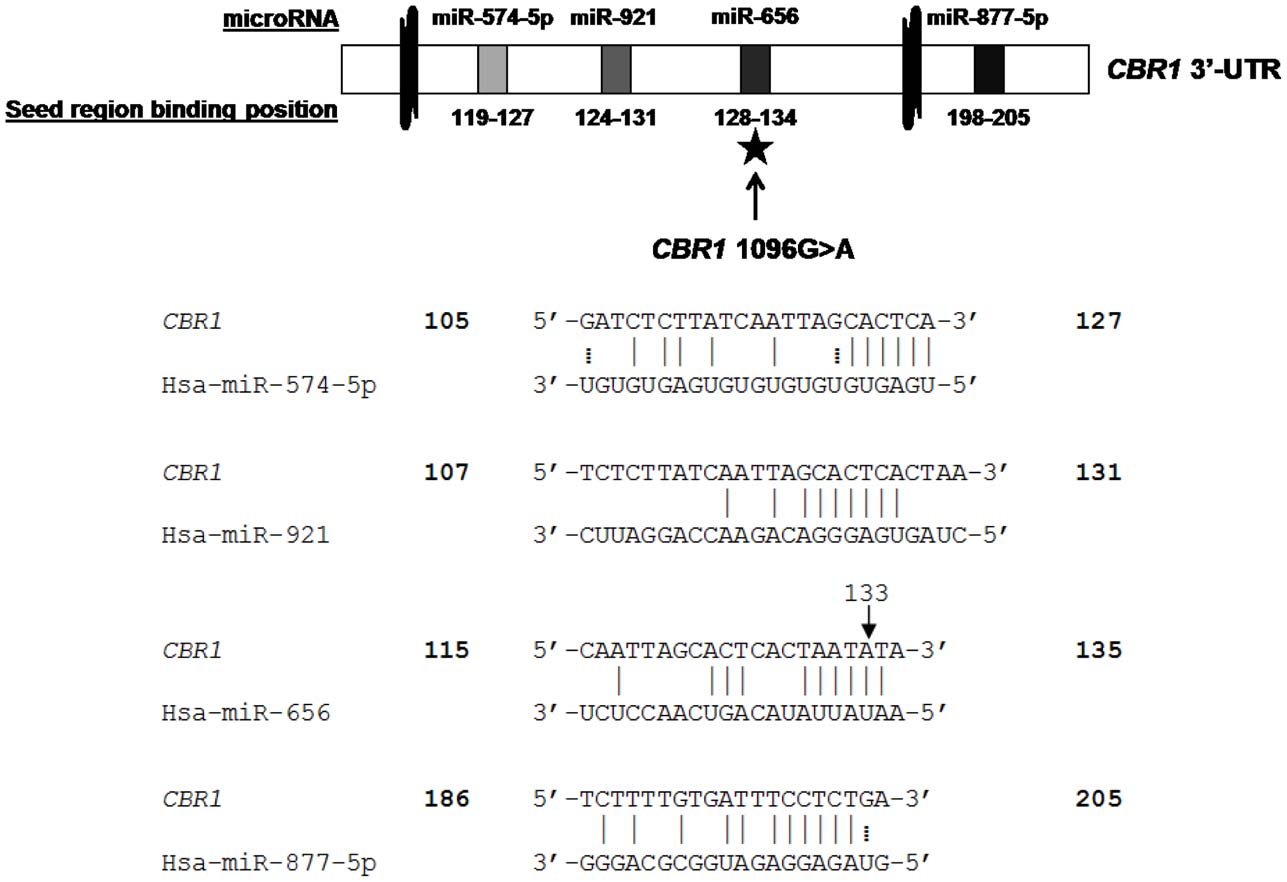
Schematic diagram of the *CBR1* 3′-UTR. Potential miRNA binding sites on the 3′-UTR of *CBR1* and their relative locations are indicated by shaded regions. The location of the *CBR1* 1096G>A polymorphism is designated with a star in the diagram and by an arrow in the sequence alignment.

PITA, TargetScan, Microcosm Targets, RNAhybrid, and PolymiRTS were used in our computational screening for human microRNA antisense matches of the *CBR1* 3′-UTR. Each algorithm produced several potential candidate interactions, with the exception of PolymiRTS which is a specialized algorithm that matches microRNA sequences to known 3′-UTR SNPs. Interestingly, PolymiRTS identified a single microRNA, hsa-miR-656, which might bind to the 3′-UTR of *CBR1* only when the minor rs9024 A allele is present. To prioritize these candidate microRNAs, we first identified miRNAs that were predicted by more than one algorithm. Candidates were then sorted by the free energy criteria ΔG_open_, the free energy lost by opening the target site, ΔG_duplex_, the free binding energy of a miRNA to the target, and ΔΔG, the difference between ΔG_open_ and ΔG_duplex_ (Table 1). These free energy values are critical in determining which microRNA/mRNA interactions are most energetically favorable. The algorithms we used pinpointed hsa-miR-574-5p, hsa-miR-656, hsa-miR-877-5p, and hsa-miR-921 as the most viable candidates for interacting with the 3′-UTR of *CBR1* (Figure 1).

**Table 1:** Potential human miRNAs that target the *CBR1* 3′-UTR (G allele/A allele).

Free energy predictions from the PITA algorithm						
microRNA	▵G_duplex_		▵G_open_		▵▵G	
	G	A	G	A	G	A
hsa-miR-574-5p	−24.49	−24.49	−6.54	−5.41	−17.94	−19.07
hsa-miR-877-5p	−19.4	−19.4	−12.75	−13.33	−6.64	−6.06
hsa-miR-921	−11.6	−5.8	−5.92	−8.04	−5.67	2.24
hsa-miR-656	N/A	−10	N/A	−5.05	N/A	−4.94

▵G values expressed in kcal/mol

### Hsa-miR-574-5p and hsa-miR-921 bind differentially to *CBR1* 3′-UTR constructs

**Figure 2:**
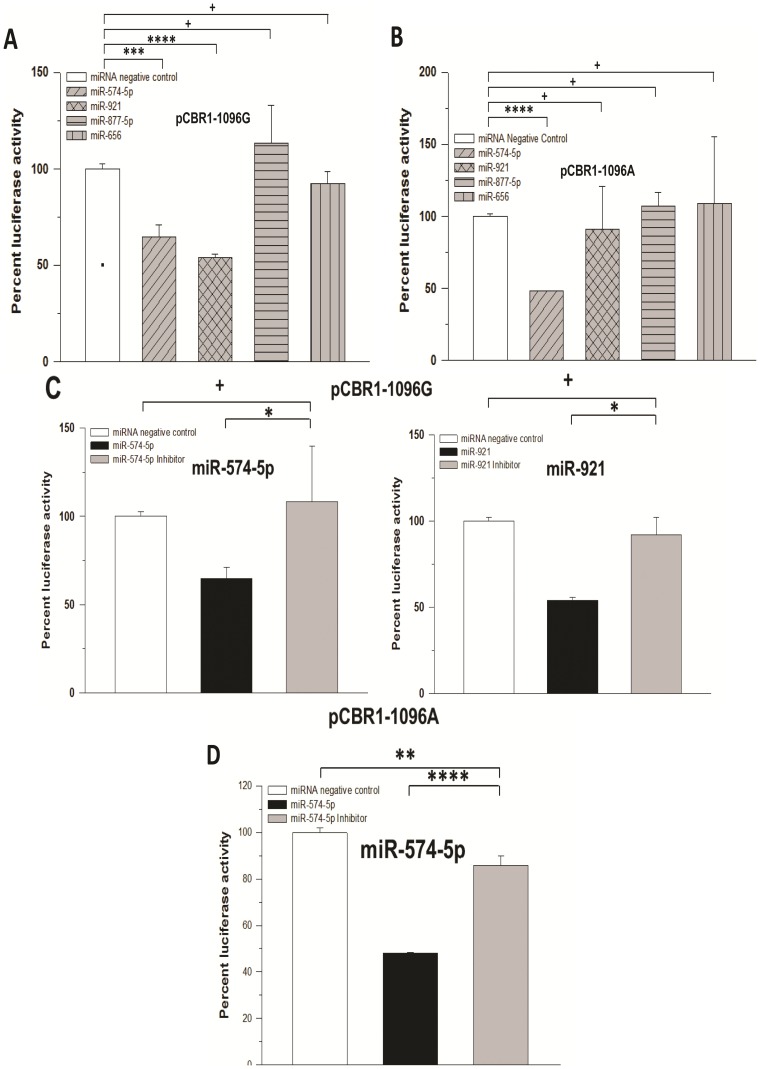
Luciferase activity in CHO cells transfected with various microRNA mimics. Luciferase activities in pCBR1-1096G (A) and pCBR1-1096A (B) constructs transfected with miRNA mimics in the presence (C) or absence (D) of specific miRNA inhibitors were measured as light intensity with a dual-luciferase assay. For each construct, normalized luciferase activities were expressed relative to the values from control transfections (miRNA negative control mimic). Data represent the mean±S.D. from three independent experiments performed in triplicate. *: *p*<0.05, **: *p*<0.01; ***: *p*<0.001; ****: *p*<0.0001; +: *p*>0.05 (Student's t-test).

We employed dual-luciferase assays to evaluate the binding of miRNA candidates to two full-length *CBR1* 3′-UTR constructs identical except for the rs9024 polymorphic site (pCBR1-1096G and pCBR1-1096A). We found that CHO cells co-transfected with pCBR1-1096G and hsa-miR-574-5p or hsa-miR-921 exhibited decreased luciferase activity compared to a miRNA negative control mimic, 35% and 46%, respectively, which suggested a significant interaction between these miRNAs and the 3′-UTR of *CBR1*. Meanwhile, transfection of hsa-miR-656 or hsa-miR-877-5p resulted in no change in luciferase activity. Additionally, hsa-miR-656 or hsa-miR-877-5p failed to significantly alter luciferase activity when paired with the pCBR1-1096A construct (Figure 2A). On the other hand, co-transfection of hsa-miR-574-5p and hsa-miR-921 with pCBR1-1096A resulted in differential regulation of luciferase activity. That is, co-transfection of hsa-miR-921 with the pCBR1-1096A construct failed to produce a significant decrease in luciferase activity while hsa-miR-574-5p decreased luciferase activity by 52% (*p*<0.0001), representing a≈1.5-fold stronger binding interaction compared to its activity with pCBR1-1096G (Figure 2B). Co-transfection of specific hairpin inhibitors for hsa-miR-574-5p was sufficient to significantly rescue the luciferase activities of the pCBR1-1096G and pCBR1-1096A constructs (Figure 2C and 2D). Similar experiments with specific hairpin inhibitors for hsa-miR-921 showed rescue of luciferase activity for the pCBR1-1096G construct (Figure 2C).

### Hsa-miR-574-5p and hsa-miR-921 regulate CBR1 protein expression in human lymphoblastoid cell lines

**Figure 3:**
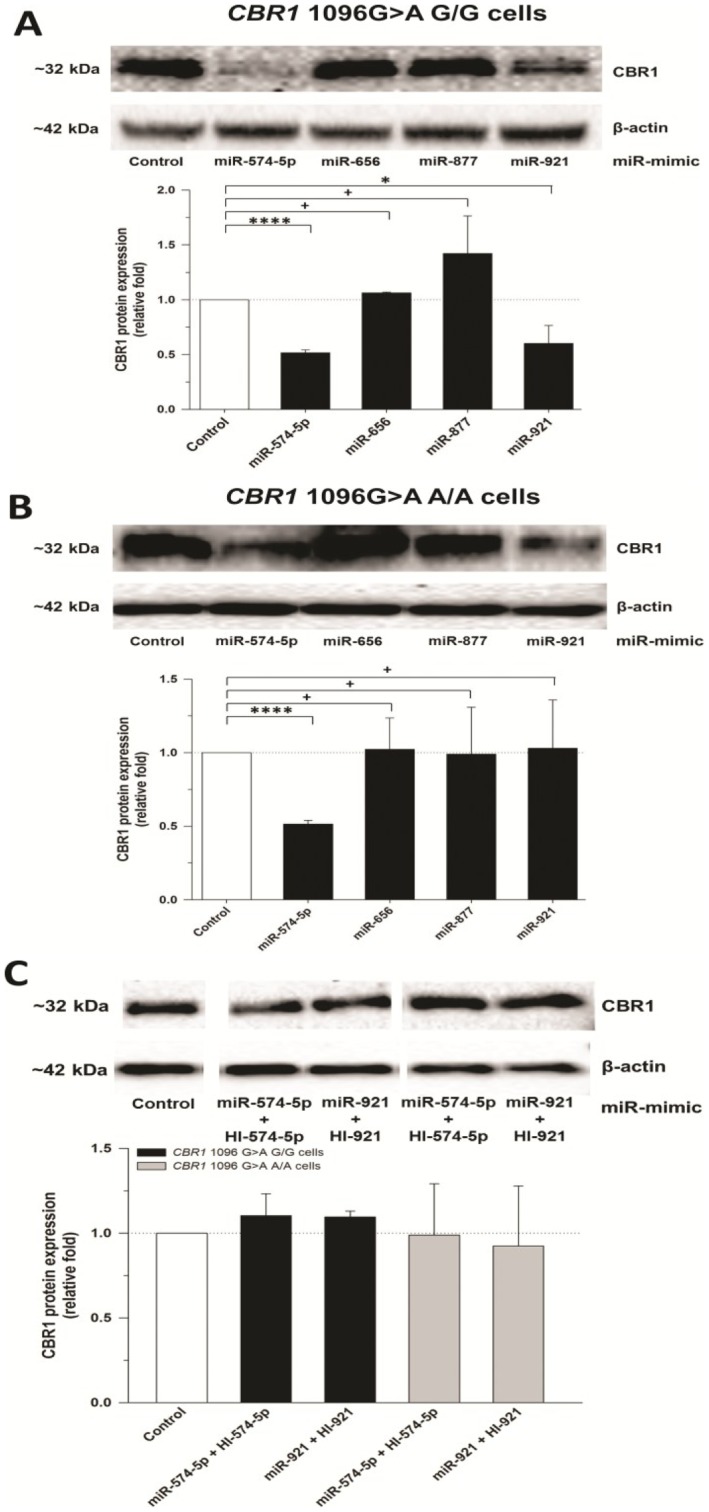
CBR1 protein expression in human lymphoblastoid cells transfected with microRNAs. β-actin-normalized CBR1 levels in lymphoblastoid cell lines with homozygosis for the *CBR1* 1096G>A SNP G allele (A) and the A allele (B) were interpolated from calibration curves of recombinant CBR1. Experiments were repeated in the presence of specific miRNA inhibitors (C). Shown first are representative western blots of CBR1 and β-actin protein. Data represent the mean±S.D. from three independent experiments performed in duplicate. ****: *p*<0.0001; +: *p*>0.05 (Student's t-test).

The results in CHO cells showed that hsa-miR-574-5p interacts with *CBR1* 3′-UTR constructs carrying the G or the A allele for rs9024 while hsa-miR-921 selectively binds only to the *CBR1* 3′-UTR construct carrying the G allele. To test the effect of these microRNAs in human cells we chose to utilize lymphoblastoid cell lines from the HapMap Project with known rs9024 (*CBR1* 1096G>A) genotype status. We have shown that *CBR1* 1096G>A genotype status impacts *CBR1* mRNA and CBR1 activity in lymphoblastoid cell lines [11]. Therefore, these cell lines represent a useful model to further investigate the effect of rs9024 genotype status on miRNA/*CBR1* interactions. Here, we utilized lymphoblastoid cells from two from donors with homozygosis for the major G allele (GM 16688 and GM 10857) and two from individuals with homozygosis for the minor A allele (GM 10853 and GM 17240). Lymphoblastoid cells homozygous for the major rs9024 G allele treated with hsa-miR-574-5p or hsa-miR-921 showed a 48% (*p*<0.0001) and 40% (*p*<0.05) reduction in CBR1 protein expression, respectively (Figure 3A). Of note, in lymphoblastoid cells homozygous for the minor rs9024 A allele, treatment with miR-574-5p reduced CBR1 protein expression by 49% (*p*<0.0001), while miR-921 incubation had no significant effect (Figure 3B). Consistent with their lack of interaction with *CBR1* 3′-UTR reporters in CHO cells, miR-656 and miR-877 failed to induce significant changes in CBR1 protein levels in lymphoblastoid cells homozygous for either rs9024 G or A alleles (Figure 3A and 3B). In both cell lines, specific miRNA inhibitors were capable of completely rescuing CBR1 protein expression (Figure 3C).

### Kinetic analysis

**Figure 4:**
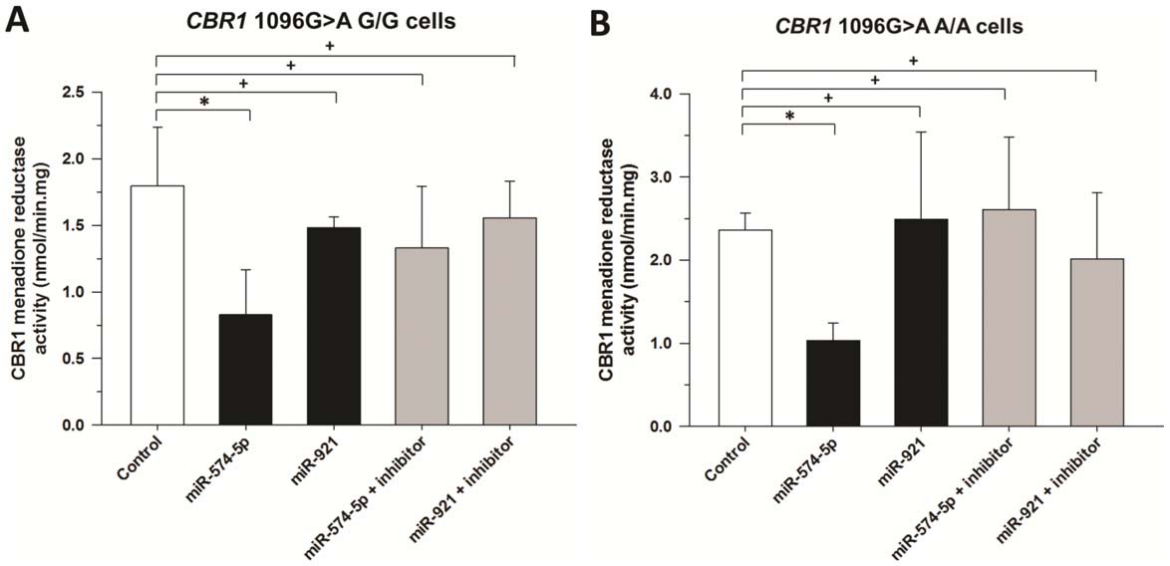
CBR1 activity in lymphoblastoid cells for the substrate menadione. Maximal CBR1 activities for the substrate menadione were determined in human lymphoblastoid cells with homozygosis for the *CBR1* 1096G>A SNP G allele (A) and the A allele (B). Enzymatic activity was assayed in triplicate. *: *p*<0.05; +: *p*>0.05.

Cytosols from lymphoblastoid cells transfected with miRNA mimics or miRNA mimics plus inhibitors were incubated with 250 µM menadione, a prototypical CBR1 substrate. CBR1 activity was significantly decreased in lymphoblastoid cells transfected with hsa-miR-574-5p (0.83±0.34 nmol/min.mg; 54% decrease; *p* = 0.04) and moderately reduced by hsa-miR-921 (1.48±0.08 nmol/min.mg; 18% decrease; *p* = 0.29), whereas co-transfection of hsa-miR-574-5p (1.33±0.46 nmol/min.mg) and hsa-miR-921 (1.55±0.27 nmol/min.mg) inhibitors rescued CBR1 menadione activity (Figure 4A). In agreement with the pattern of inhibition of CBR1 protein expression in lymphoblastoid lines homozygous for the minor rs9024 A allele, miR-574-5p diminished CBR1 activity by 56% (*p* = 0.001) while miR-921 exerted no significant impact on CBR1 activity (*p* = 0.84) (Figure 4B).

### Hsa-miR-574-5p- and hsa-miR-921-mediated regulation of *CBR1* involves mRNA degradation

**Figure 5:**
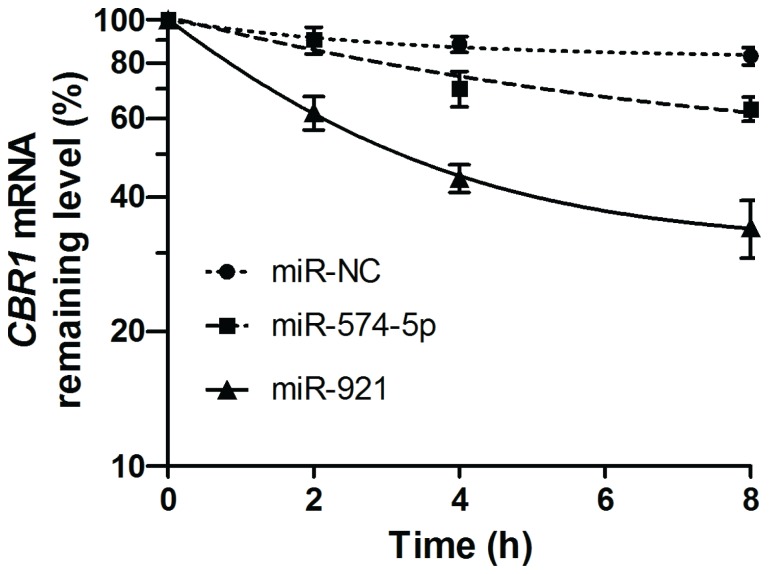
*CBR1* RNA degradation. Relative *CBR1* mRNA levels were determined with a comparative quantitation method in cells transfected with miRNA negative control (hsa-miR-NC), hsa-miR-574-5p, or hsa-miR-921. Individual *β-actin* mRNA levels were used as normalizers. Results shown are mean±SD from triplicate exposures.

To elucidate the role of mRNA degradation in hsa-miR-574-5p- and hsa-miR-921-mediated post-transcriptional regulation of *CBR1*, we conducted RNA degradation experiments. The lymphoblastoid cell line GM 10857 was co-transfected with hsa-miR-574-5p and hsa-miR-921 for 48 hours and then treated with actinomycin D, a potent *de novo* transcription inhibitor. Inspection of apparent RNA half-lives demonstrated that both hsa-miR-574-5p (*CBR1* mRNA t_1/2_>8 hours, but<<*CBR1* mRNA t_1/2_ of miRNA negative control) and hsa-miR-921 (*CBR1* mRNA t_1/2_≈3.5 hours) accelerated decay of *CBR1* mRNA compared to the miRNA mimic negative control (Figure 5).

### Hsa-miR-574-5p is expressed in human liver and heart tissues

**Figure 6:**
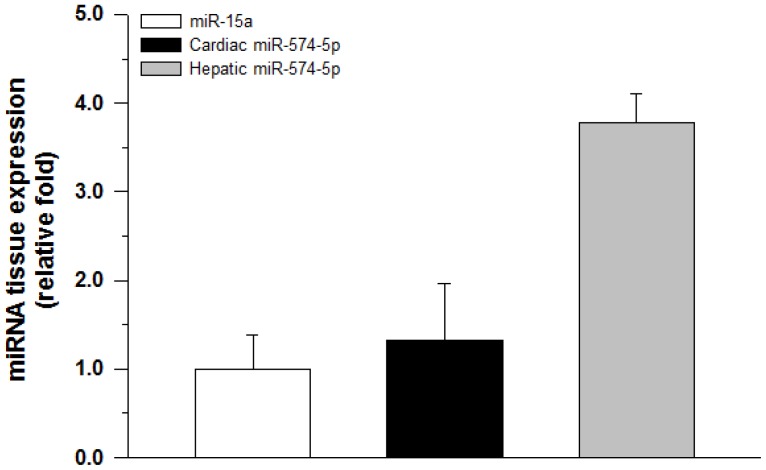
miR-574-5p expression in human tissues. Relative miR-574-5p miRNA levels were determined with a comparative quantitation method in total RNA extracted from human heart and human liver samples. Individual U6 expression levels were used as normalizers. Results shown represent the mean±SD expression levels of triplicate experiments from 4 tissue samples of each tissue type.

Direct sequencing of real-time PCR products confirmed the specific amplification of our positive control hsa-miR-15a as well as hsa-miR-574-5p. However, commercial primers for hsa-miR-921 failed to amplify the correct mature miRNA sequence. Quantitative RT-PCR gene expression detection of hsa-miR-15a and hsa-miR-574-5p in total RNA isolated from 4 human liver and 4 heart samples showed that both miRNAs were present in all samples (Figure 6). U6-normalized expression levels of hsa-miR-574-5p were favorable compared to individual hsa-miR-15a expression levels in liver (3.8±0.3 relative fold) and heart (1.3±0.7 relative fold).

## Discussion

Previously, we reported that hepatic and cardiac CBR1 expression is highly variable [11], [12]. We also pinpointed a SNP in the 3′-UTR of *CBR1*, rs9024 (*CBR1* 1096G>A), that has a functional impact on *CBR1* mRNA and CBR1 protein expression as well as CBR activity [11]. The physiologic origin of this genotype/phenotype association has not yet been elucidated. Here, we posited that rs9024 may affect microRNA regulation of *CBR1* due to its proximity to several bioinformatically-predicted miRNA binding sites on the *CBR1* 3′-UTR. This interplay could play a significant role in the observed variability of *CBR1* expression.

The current study was designed to identify potential miRNA interactions with the 3′-UTR of the *CBR1* gene and assess the influence of rs9024 on miRNA-mediated post-transcriptional regulation of *CBR1* (Table 2). Bioinformatic predictions led us to examine hsa-miR-574-5p, hsa-miR-656, hsa-miR-877-5p, and hsa-miR-921 as prime *CBR1* binding candidates. We demonstrated that miR-574-5p and hsa-miR-921 both influence CBR1 protein expression in human cell lines with homozygosis for the major *CBR1* 1096G>A G allele (Figure 3A and Table 2). On the other hand, in cell lines homozygous for the minor A allele only hsa-miR-574-5p produced significant decreases in CBR1 protein levels (Figure 3B and Table 2). These reductions in CBR1 protein levels corresponded to substantially diminished maximal CBR1 menadione activity. Of note, a priori it was anticipated that hsa-miR-656 would demonstrate no binding capacity for the rs9024 G allele, but a significant ability to interact with the *CBR1* 3′-UTR in its rs9024 polymorphic state (A allele) due to the establishment of a novel binding site by the single base-pair substitution. Contrary to these predictions, hsa-miR-656 exhibited no effect on CBR1 expression or activity. It appears that this seemingly surprising result can be explained by the energetically unfavorable free energy conditions that would accompany the binding of hsa-miR-656 to *CBR1* (Table 1).

**Table 2:** Comprehensive *CBR1*/miRNA results summary.

		G/G/A/A		
Bioinformatic predictions	miR-574-5p	miR-877-5p	miR-921	miR-656
CHO cells	35% ↓/52% ↓	NC/NC	46% ↓/NC	N/A/NC
Lymphoblastoid protein	48% ↓/49% ↓	NC/NC	40% ↓/NC	N/A/NC
Lymphoblastoid activity	54% ↓/56% ↓	NC/NC	18% ↓/NC	N/A/NC

Although the SNP rs9024 does not directly alter the 3′-UTR target binding sites of hsa-miR-574-5p (position 120) or hsa-miR-921 (position 125) seed regions, it is notable that both binding sites are proximal to the polymorphic site at position 133 (Figure 1). The juxtaposition of the SNP and these target sites may alter the binding of these miRNAs by affecting factors such as ΔG_open_, the free energy lost by opening the target site and ΔG_duplex_, the free binding energy of a miRNA to the target. Indeed, Mishra et al. found that a SNP in the 3′-UTR of the dihydrofolatereductase gene 14 bp downstream of the hsa-miR-24 binding site led to a phenotypic change in methotrexate resistance [28]. Hu and Bruno conducted a genome-wide study of SNPs in genes known to be targeted by miRNAs and discovered that SNPs in seed target regions as well as the entire 3′-UTR of miRNA target genes were under significantly greater negative selection compared to non-miRNA target genes. Moreover, they found that local RNA sequences ∼67 nucleotides symmetrically surrounding miRNA target sites were under moderate strong selection [29]. It is possible that the proximity of hsa-miR-574-5p and hsa-miR-921 binding sites, 13 bp and 8 bp, respectively, upstream of the polymorphic site, is the underlying cause of the changes observed in free energy predictions between the two rs9024 variants of the *CBR1* 3′-UTR. The accessibility of the *CBR1* message to miRNA binding partners may impart differential miRNA regulation of the *CBR1* gene leading to downstream impacts on CBR1 protein expression and activity. We hypothesize that this phenomenon contributes to interindividual variability in *CBR1* mRNA, CBR1 protein expression and CBR activity which could have major implications in the metabolism of a host of endogenous and xenobiotic substrates.

Importantly, hsa-miR-574-5p and hsa-miR-921 were co-expressed with *CBR1* in human liver and heart tissue. Previous studies have reported that CBR1 is the primary anthracycline reductase in liver [11], [13] and participates in the formation of cardiotoxic alcohol metabolites in the heart [12], [30], [31]. MicroRNAs may play a significant role in the regulation of *CBR1*, although it is important to recognize that they represent one part of a complex array of contributing regulatory factors [12], [24], [32]. Our pilot work detecting the presence of miR-574-5p in liver and heart tissues suggests that there is significant interindividual variability in the expression of this miRNA in these tissues (Figure 6). Future work will attempt to characterize the extent of interindividual variability in the expression of hsa-miR-574-5p and hsa-miR-921 in human tissues in tandem with *CBR1* 1096G>A genotype and assess their combined impact on CBR1 phenotypic variability.
